# Supplementary material for: Resveratrol Alleviated Oxidative Damage of Bovine Mammary Epithelial Cells via Activating SIRT5-IDH2 Axis
Source: Antioxidants (Basel). 2025 Sep 26;14(10):1171. doi: 10.3390/antiox14101171 (PMC12562252; doi:10.3390/antiox14101171)
Supplement: Supplementary file 1 [file antioxidants-14-01171-s001.zip › antioxidants-3850408-supplementary.pdf]

antioxidants

# Supplementary files

*Effect of $H_2O_2$ on the viability of bMECs*

In this study, $H_2O_2$ was used as an oxidative stress agent. To determine appropriate $H_2O_2$ concentration to treat cells, bMECs were exposed to various concentrations of $H_2O_2$ (0, 100, 200, 300, 400, 500, 600, 700, and 800 μM) for 24 h, respectively. The viability of bMECs was evaluated by CCK-8 method. As illustrated in Figure S1, $H_2O_2$ reduced the viability of bMECs in a concentration-dependent manner. Specifically, at 100 μM concentration, $H_2O_2$ decreased significantly cell viability ($P<0.01$), while cell viability reduced significantly about 50 % at 500 μM ($P<0.01$). Considering the effect of $H_2O_2$ on oxidative stress, 500 μM $H_2O_2$ was chosen to treat cells in the subsequent study.

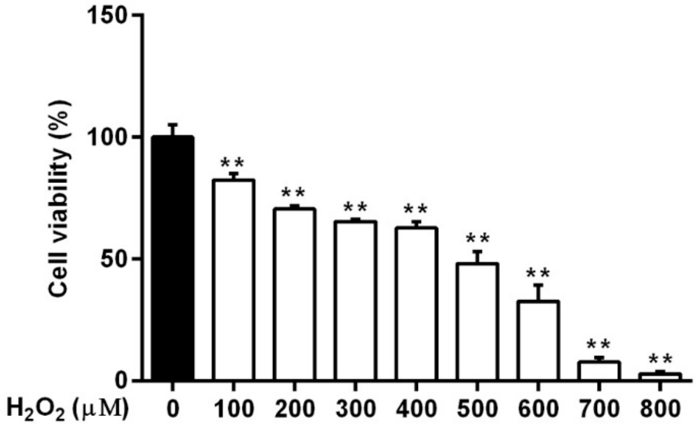


***Figure S1. Effect of $H_2O_2$ on the viability of bMECs.*** $H_2O_2$ was used as the stress agent. When bMECs were cultured reaching the confluent extent of 80~90 %, cells were exposed to various concentrations of $H_2O_2$ (0, 100, 200, 300, 400, 500, 600, 700, and 800 μM) for 24 h, respectively. The viability of bMECs was evaluated by CCK-8 method. Data are list as mean ± SEM of at least three independent experimental determinations, and statistically analyzed by two-tailed followed by unpaired t-test. **$P < 0.01$ vs. 0 μM $H_2O_2$.

*Effect of RES on the viability of bMECs*

In this study, RES was used as an antioxidant. To determine appropriate RES concentration to treat cells, bMECs were exposed to various concentrations of RES (0, 10, 20, 30, 40, 50, 60, 70, and 80 μM) for 24 h, respectively. The viability of bMECs was evaluated by CCK-8 method. As illustrated in Figure S2, RES (10, 30, and 40 μM) did not affect the viability of bMECs, while RES (50, 60, 70, and 80 μM) reduced obviously the viability of bMECs in a concentration-dependent manner. Exceptionally, at 20 μM concentration, RES enhanced significantly the viability of bMECs ($P<0.05$).

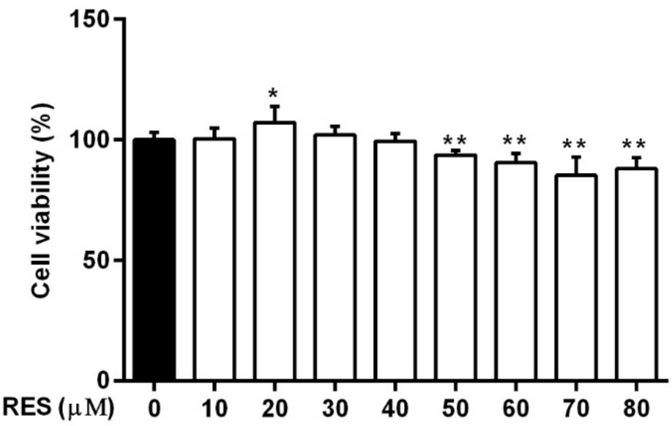


***Figure S2. Effect of RES on the viability of bMECs.*** RES was used as an antioxidant. When bMECs were cultured reaching the confluent extent of 80~90 %, cells were exposed to various concentrations of RES (0, 10, 20, 30, 40, 50, 60, 70, and 80 μM) for 24 h, respectively. The viability of bMECs was evaluated by CCK-8 method. Data are list as mean ± SEM of at least three independent experimental determinations, and statistically analyzed by two-tailed followed by unpaired t-test. *P < 0.05, **P < 0.01 vs. 0 μM RES.

*Effect of RES and $H_2O_2$ co-treatment on the viability of bMECs*

bMECs were exposed to various concentrations of RES (0, 10, 20, 30, 40, and 50 μM) and 500 μM $H_2O_2$ for 24 h, respectively. The viability of bMECs was evaluated by CCK-8 method. As illustrated in Figure S3, RES (10, 20, 30, 40, and 50 μM) enhanced the viability of bMECs in a concentration-dependent manner, compared with 500 μM $H_2O_2$ treatment. While 500 μM $H_2O_2$ reduced obviously the viability of bMECs, compared with 0 μM $H_2O_2$. Considering that RES treatment alone did not affect cell viability, while co-treatment could maximize cell viability, 40 μM RES was chosen to treat cells in this study.

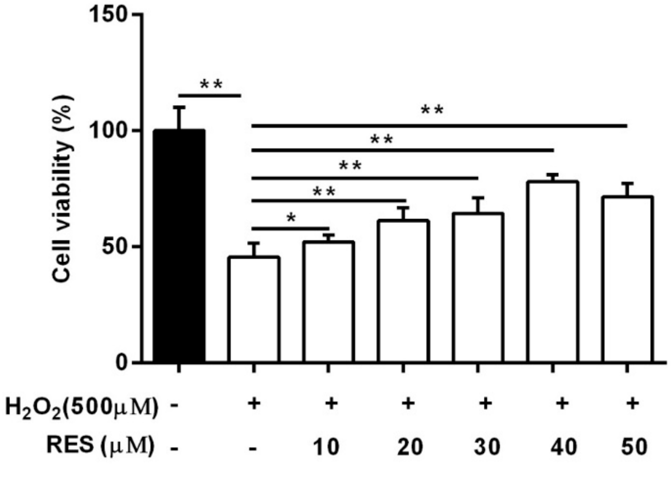


***Figure S3. Effect of RES and $H_2O_2$ co-treatment on the viability of bMECs.*** When bMECs were cultured reaching the confluent extent of 80~90 %, cells were exposed to various concentrations of RES (0, 10, 20, 30, 40, and 50 μM) and 500 μM $H_2O_2$ for 24 h, respectively. The viability of bMECs was evaluated by CCK-8 method. Data are list as mean ± SEM of at least three independent experimental determinations, and statistically analyzed by two-tailed followed by unpaired t-test. *P < 0.05, **P < 0.01 vs. 0 μM RES and $H_2O_2$, or 500 μM $H_2O_2$.

*Effect of RES on LDH release in bMECs*

In this study, we detected the LDH release in the supernatant of cell culture. As shown in Figure S4, compared to CT group, the LDH release was enhanced obviously in

$H_2O_2$ group. After RES treatment, the LDH release was reduced markedly in RES+$H_2O_2$ co-treatment group (RESH), compared with $H_2O_2$ group. Inhibiting SIRT5 using NAM (an inhibitor for SIRT5), the LDH release was increased significantly in RESNH group, compared with RESH group. Interestingly, the LDH release was increased obviously in NH group, compared with $H_2O_2$ group. These results suggested that RES reduced the LDH release related to activation of SIRT5.

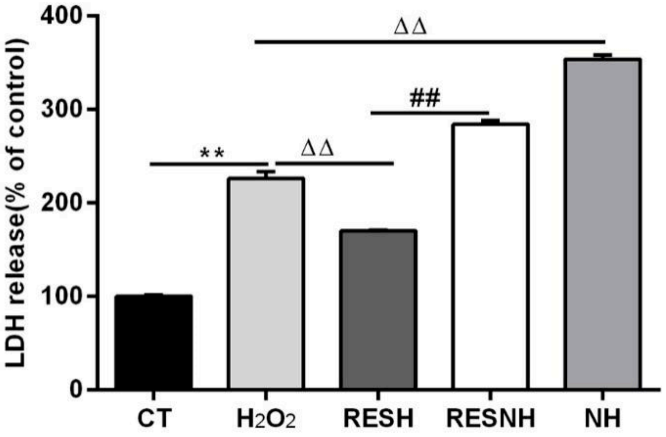


***Figure S4. Effect of RES on LDH release in bMECs.*** When bMECs were cultured reaching the confluent extent of 80~90 %, which were randomly divided into five groups. bMECs in the CT group were cultured in basal medium. bMECs in $H_2O_2$ group were exposed to 500 μM $H_2O_2$ for 24 h. bMECs in the RESH group were exposed to 500 μM $H_2O_2$ and 40 μM RES for 24 h. bMECs in RESNH group were exposed to 500 μM $H_2O_2$, 40 μM RES and 50 μM NAM for 24 h. bMECs in NH group were exposed to 500 μM $H_2O_2$ and 50 μM NAM for 24 h. The LDH release in the supernatant of cell culture was determined by commercial LDH kits. Data are list as mean ± SEM of at least three independent experimental determinations, statistically analyzed by a one-way analysis of variance. $^{**}P < 0.01$ vs. CT group, $\Delta\Delta P < 0.01$ vs. $H_2O_2$ group, $\#\#P < 0.01$ vs. RESH group.

*Effect of SIRT5 on LDH release in bMECs*

To further demonstrate the correlation between RES reducing LDH release and SIRT5, we used $H_2O_2$ to treat SIRT5 overexpression cells or cells treatment with NAM. As list in Figure S5, compared to CT group, the LDH release was enhanced obviously in $H_2O_2$ group. The LDH release in SIRT5 overexpression cells was reduced markedly in SOH group, compared with $H_2O_2$ group. Conversely, inhibiting SIRT5 using NAM (an inhibitor for SIRT5), the LDH release was increased significantly in SONH group, compared with SOH group. These results indicated that RES reduced the LDH release associated with SIRT5.

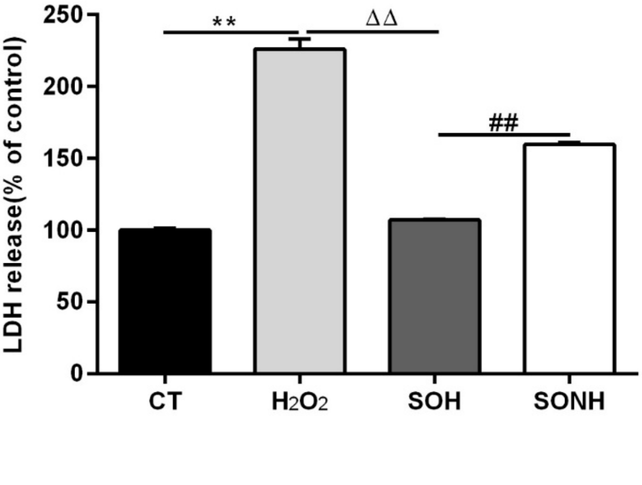

***Figure S5. Effect of SIRT5 on LDH release in bMECs.*** When bMECs and SIRT5 overexpression cells were cultured reaching the confluent extent of 80~90%, which were randomly divided into four groups. bMECs in CT group were seed in basal medium. bMECs in $H_2O_2$ group were exposed to 500 μM $H_2O_2$ for 24 h. SIRT5 overexpression cells in SOH group were exposed to 500 μM $H_2O_2$ for 24 h. SIRT5 overexpression cells in SONH group were exposed to 500 μM $H_2O_2$ and 50 μM NAM for 24 h. The LDH release in the supernatant of cell culture was determined by commercial LDH kits. Data are list as mean ± SEM of at least three independent experimental determinations, and statistically analyzed by a one-way analysis of variance. **P < 0.01 vs. CT group, ΔΔP < 0.01 vs. $H_2O_2$ group, ##P < 0.01 vs. RESH group.

*Effect of NAM on the viability of bMECs*

In this study, nicotinamide (NAM) was chosen as an inhibitor for SIRT5. To determine appropriate NAM concentration to treat cells, bMECs were exposed to various concentrations of NAM (0, 10, 20, 30, 40, 50, 60, 70, and 80 μM) for 24 h, respectively. The viability of bMECs was evaluated by CCK-8 method. As illustrated in Figure S6, NAM (10, 20, 30, 40, and 50 μM) had no effect on the viability of bMECs, while NAM (60, 70, and 80 μM) reduced obviously the viability of bMECs in a concentration-dependent manner. Therefore, 50 μM NAM was chosen to treat cells in this study.

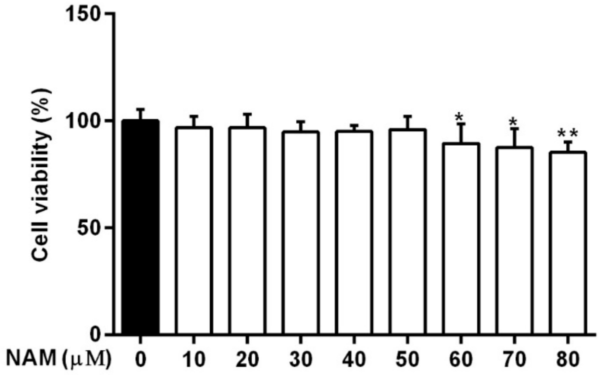


***Figure S6. Effect of NAM on the viability of bMECs.*** NAM was chosen as an inhibitor for SIRT5. When bMECs were cultured reaching the confluent extent of 80~90 %, cells were exposed to various concentrations of NAM (0, 10, 20, 30, 40, 50, 60, 70, and 80 μM) for 24 h, respectively. The viability of bMECs was evaluated by CCK-8 method. Data are list as mean ± SEM of at least three independent experimental determinations, and statistically analyzed by two-tailed followed by unpaired t-test. *P < 0.05, **P < 0.01 vs. 0 μM NAM.
